# Assessment of Pulp Stones Among Western Saudi Populations: A Cross-Sectional Study

**DOI:** 10.7759/cureus.46056

**Published:** 2023-09-27

**Authors:** Mahir A Mirah, Arwa Bafail, Sameer Shaheen, Abdulmajeed Baik, Basim Abu Zaid, Abdulaziz Alharbi, Omar Alahmadi

**Affiliations:** 1 Restorative Dental Sciences Department, College of Dentistry, Taibah University, Madinah, SAU; 2 Oral Basic and Clinical Sciences Department, College of Dentistry, Taibah University, Madinah, SAU; 3 Dentistry, Taibah University, Madinah, SAU

**Keywords:** madinah, prevalence, pulp calcification, cbct, pulp stone

## Abstract

Background: The term "pulp stones" refers to conditions of calcification that can appear in any area of the dental pulp. This study aims to assess the prevalence of pulp stones and examine whether there is any connection between their occurrence and factors such as patient age, gender, tooth location, presence of decay, or dental restorations in a western Saudi subpopulation based on cone-beam computed tomography (CBCT).

Methodology: 500 patients were randomly selected from the College of Dentistry, Taibah University, Madinah, Saudi Arabia, database. The study involved evaluating 2998 teeth in coronal, axial, and sagittal planes by four dental peer examiners observing and recording data (sequentially and simultaneously) that included whether pulp stones were present or absent, the teeth group (anterior, premolars, and molars), tooth location (maxillary or mandibular), presence or absence of restoration, presence or absence of caries, and the patient's gender. Chi-square tests were utilized for statistical analysis, and a significance level of p-value ≤0.05 was established.

Results: Out of 500 individuals (250 females and 250 males), only 130 individuals (26%) and 278 teeth out of 2998 (9.2%) had pulp stones, with no significant statistical correlation identified between the presence of pulp stones and gender. (P = 0.459). Molars exhibited the greatest incidence of pulp stones (238; 85.6%), followed by anterior teeth (30; 10.8%), and premolars had the lowest prevalence (10; 3.6%). Teeth that showed a higher occurrence of pulp stones were associated with caries (74; 26.6%) and restorations (58; 20.9%). The association between pulpal stone and periodontal involvement was seldom significant (0.7%). A significant association was found between the presence of pulp stones and nonintact teeth (P<0.001). There was a statistically significant difference in the distribution of pulp stones between females and males in the maxillary and mandibular (74.7% and 57.3%, respectively) (P = 0.002). Additionally, the frequency of pulp stones was statistically significant when comparing the left and right sides (P<0.001) (48.9% and 51.1%, respectively).

Conclusion: Understanding the prevalence and distribution of pulp stones is crucial for dentists and endodontists, as it assists practitioners in devising an appropriate treatment plan for affected teeth that require root canal therapy. One-fourth of the Madinah population was confirmed to have pulp stones, with a higher incidence in molars, caries, and restored teeth. No difference was found between its occurrence and gender. The high prevalence is exhibited in individuals between 45 and 54 years old. However, further studies with equal patient distribution are needed to confirm this observation.

## Introduction

In modern dentistry, cone-beam computed tomography (CBCT) has emerged as a valuable diagnostic tool as it surpasses the restrictions of traditional radiographs by providing detailed anatomical information from multiple views, enabling dentists to diagnose and plan treatments more accurately. CBCT offers several significant benefits, including precision, reliability, high-quality resolution, and the ability to produce three-dimensional images without overlapping structures. Due to these advantages, CBCT is increasingly being used in dentistry; it appears that the usage of CBCT for clinical examination of pulp stones is about to become widespread [1].

Pulp stones are areas of calcification that can be present in any region of the dental pulp, whether in primary or permanent teeth; they can also be located in intact, compromised, and potentially even in teeth that have not yet emerged [2,3]. During radiographic evaluation, pulp stones can be seen as radiopaque masses in different shapes and sizes [4]. Structurally, three distinct types of pulp stones can be distinguished: "true pulp stones," which are composed of regular tubular dentin and are surrounded by odontoblasts; "false pulp stones," which form from mineralized degenerating pulp cells and "amorphous" pulp stones, which have a less organized shape than false pulp stones [5]. Studies have indicated that pulp stones can occur in the radicular pulp but are more common in the coronal region [6]. When performing root canal treatment, pulp stones can increase the risk of treatment complications such as perforations, loss of tooth structure, loss of working length, or failure of the root canal treatment [7-10]. Furthermore, when pulp stones are present, especially at the curved portion of the root's apical third, they can obstruct the root canals, making it challenging to perform root canal procedures [11]. Moreover, the exact cause behind the development of pulp stones is still uncertain. However, several factors have been suggested to contribute to their formation, including calcification of the pulp, orthodontic tooth movement, aging, periodontal disease, deep caries, calcifying nanoparticles, bacterial infection, systemic diseases, restorations, and genetic predisposition [12-15]. A significant association has been observed between pulp stones and systemic diseases, such as diabetes, heart disease, cholelithiasis, and renal lithiasis [16].

The prevalence of pulp stones and their connection to diabetes mellitus and cardiovascular diseases in Saudi Arabia were investigated in a study. A total of 73 patients with cardiovascular disorders were included in the study, of which 24 (32.87%) had pulp stones. Similarly, out of 76 patients with diabetes mellitus, 18 (23.68%) had pulp stones [16]. A study done by Al-Shamrani and Al-Nazhan on the Saudi Arabian population revealed that the incidence of pulp stones was 859 teeth (10.2%) [17]. According to Tamse et al., pulp stones were more significant in molars than premolars, and females were more frequently affected than males. However, Patil et al. reported that males from the northern region of Saudi Arabia had an increased incidence of pulp stones compared to females [18,19]. Dental specialists may encounter procedural difficulties during endodontic treatments when pulp stones are present. As a result, understanding the potential factors, frequency, and pattern of distribution of pulp stones can be beneficial in anticipating challenges during treatment. Thus, this research aimed to evaluate the prevalence of pulp stones and investigate any potential links between their occurrence and relevant factors such as age, gender, tooth location, caries, and restorations in a subpopulation of Western Saudi Arabia, mainly by using CBCT.

## Materials and methods

This study is a retrospective analysis performed on 500 CBCT images of Saudi patients (250 males and 250 females) who pursued dental treatment for various reasons, such as missed canals, oral surgery, and dental implants. The study was conducted at Taibah University's College of Dentistry Radiology Department, Madinah, in the Western region of Saudi Arabia. Six age groups are included in the study: patients aged 18-24, 25-34, 35-44, 45-54, 55-64, and those older than 65. Images that were unclear or distorted, teeth that had received endodontic therapy, teeth with crowns or posts, root resorption, third molars, and deep carious lesions or restorations that obscured the pulp chamber were excluded. The final sample comprised 500 CBCT images, which were examined to determine the relationship between dental factors and pulp stones. The data observed and recorded includes pulp stone (presence or absence), teeth group (anterior, premolars, and molars), location of tooth (maxillary or mandibular), restoration (presence or absence), caries (presence or absence), and patient's gender. The CBCT images used in the study were obtained and examined at the Radiology Department of Taibah University's College of Dentistry using the CBCT machine (Carestream CS 9300) to obtain all CBCT scans utilized in this study. The parameters of the machine were set to 90 kVp, 4 mA, and 8 seconds of acquisition time for the "Medium Patient" set. For the "Large Patient" set, the settings were 90 kVp, 5 mA, and 8 seconds. When the CBCT was for mandible only, the settings were 90 kVp, 5 mA, and 8 seconds of acquisition time.

The data were transferred from the Excel sheet to IBM Corp. Released 2019. IBM SPSS Statistics for Windows, Version 26.0. Armonk, NY: IBM Corp for analysis. Descriptive analyses were carried out to report sample characteristics. Quantitative variables were analyzed as frequencies and percentages. A nonparametric (chi-square) test was used to compare the occurrence of pulp stones with sex, tooth types, associated factors, tooth location, tooth position, and teeth group. The level of significance was set at a p-value ≤0.05.

Descriptive analyses were carried out to report sample characteristics. Quantitative variables were analyzed as frequencies and percentages. A nonparametric (chi-square) test was used to compare the occurrence of pulp stones with sex, teeth types, associated factors, tooth location, tooth position, and teeth group. The level of significance was set at a p-value ≤0.05.

## Results

Among the 500 individuals, 130 were identified as having pulp stones, representing 26%. In addition, one or more pulp stones were identified in 278 (9.2%) teeth through the CBCT examination. Sixty-three (25.2%) males and 67 (26.8) females had pulp stones, with no significant association between gender and the presence of pulp stones (P = 0.459). The pulp stones in the teeth with carious teeth were 74 (26.6%), teeth with restoration were 58 (20.9%), and the least frequent were in teeth affected by periodontal disease (2 (0.7%)). The distribution of pulp stones according to gender is presented in Table 1.

**Table 1:** Distribution of pulp stones according to gender X^2^ = Chi-squared tests * Statistical significance difference between the variables (P<0.05)

Gender	Number of teeth examined	Number of teeth with pulp stones	Number of teeth without pulp stones	X^2^	P-value
Males	1540	124 (8.75%)	1416 (91.94%)	0.548	0.459
Females	1469	154 (11.71%)	1315 (89.51%)		
Total	3009	278 (9.23%)	2731 (90.76%)		

The study revealed a significant association between the presence of pulp stones and teeth that were not intact (P<0.001). According to the findings, there is no significant variation between males and females with respect to other risk factors (P=0.470) (Table 2).

**Table 2:** Association between the presence of pulp stones and associated factors N = Number of teeth X^2^ = Chi-squared tests * Statistical significance difference between the variables (P<0.05)

Presence of pulp stones (N=278)									
Gender		Males			Females			X^2^	P-value
		N = 124 (44.6%)			N = 154 (55.4%)			0.548	0.459
		N	%		N	%			
Associated factors	Restoration	27		21.8%	31		20.1%	2.600	0.470
	Caries	29		23.4%	45		29.2%		
	Periodontally involved teeth	0		0.0%	2		1.3%		
Tooth location	Maxillary arch N= 186	71		57.26%	115		74.67%	9.411	0.002^*^
	Right	45		63.38%	68		59.13%	1643.258^*^	<0.001^*^
	Left	26		36.62%	47		40.86%		<0.001^*^
	Mandibular arch N= 92	53		42.74%	39		25.32%	9.411	0.002^*^
	Right	31		58.49%	21		53.84%	1643.258^*^	<0.001^*^
	Left	22		41.50%	18		46.15%		<0.001^*^

According to the study, pulp stones were mostly found in molars (238, 85.6%), followed by anterior teeth (30, 10.8%), and uncommon in premolars (10, 3.6%) (Table 3).

**Table 3:** Distribution of pulp stones according to teeth group N = Number of teeth

Teeth group	Number of teeth (N=278)	Percent
Anterior	30	10.8%
Premolar	10	3.6%
Molar	238	85.6%

A higher incidence of pulp stones was found in the maxillary teeth (186, 66.9%) compared to the mandibular teeth (92, 33.1%), with a significant statistical dissimilarity between females and males (P=0.002). Finally, the cases of pulp stones on the left and right sides showed almost even distribution (136, 48.9%) and (142, 51.1%), respectively (Table 4).

**Table 4:** Distribution of pulp stones according to teeth location and position N = Number of teeth

Distribution of pulp stones according to tooth location (N=278)			
Left		Right	
136 teeth	48.9%	142 teeth	51.1%
Distribution of (pulp stones according to tooth position (N=278)			
Maxillary arch		Mandibular arch	
186 teeth	66.9%	92 teeth	33.1%

The prevalence of patients with pulp stones with age shows that 16.09% of them aged 18-24 have them. This also increased in patients aged 25-34 to 29.17%. The highest prevalence of pulp stones was found in the age group range of 45-54; 34.52% of patients had pulp stones. There was a statistically significant difference between the age and the number of patients with pulp stones (P = 0.024). However, no significant differences were found between males and females (P = 0.289).

The prevalence of teeth with pulp stones with age shows that 6.83% of patients aged 18-24 have pulp stones. This also increased in patients aged 25-34 to 10.87%. The highest prevalence of pulp stones was found in the age group range of 45-54; 12.34% of patients had pulp stones. There was a statistically significant difference between the age and the number of teeth with pulp stones (P = 0.003). However, no significant differences were found between males and females (P = 0.471) (Table 5).

**Table 5:** Distribution of pulp stones according to age N = number of teeth n = number of patients * Statistical significance difference between the variables (P<0.05)

	Patients with pulp stone				Teeth with pulp stone			
Age range	M n (%)	F n (%)	T n (%)	P-value	M N (%)	F N (%)	T N (%)	P-value
18-24	0 (0.00%)	14 (31.82%)	14 (16.09%)	*0.024	0 (0.00%)	36 (14.94%)	36 (6.83%)	*0.003
25-34	19 (31.67%)	16 (26.67%)	35 (29.17%)		28 (8.62%)	41 (13.23%)	69 (10.87%)	
35-44	22 (32.35%)	12 (22.22%)	34 (27.87%)		45 (12.03%)	21 (7.89%)	66 (10.31%)	
45-54	12 (30.00%)	17 (38.64%)	29 (34.52%)		22 (10.63%)	36 (13.69%)	58 (12.34%)	
55-64	7 (21.21%)	6 (24.00%)	13 (22.41%)		23 (13.14%)	7 (2.79%)	30 (7.04%)	
65 or over	3 (50.00%)	2 (8.70%)	5 (17.24%)		6 (16.22%)	13 (4.74%)	19 (6.11%)	
Total	63 (25.20%)	67 (26.80%)	130 (26%)		124 (8.83%)	154 (9.60%)	278 (9.24%)	

## Discussion

Pulp stones do not impede endodontic procedures; larger ones can make it difficult to access the root canal as they can block the canal and break the endodontic instruments [17]. As a result, it is advisable to obtain suitable preoperative radiographs and use ultrasonic instruments to remove pulp stones effectively. Furthermore, in some cases, patients may complain of unexplained tooth pain, which could be attributed to the compression of sensory nerves by the pulp stones [17]. Typically, the existence of pulp stones does not necessitate any intervention.

Pulp stones larger than 200 um are visible in conventional and digital extra and intraoral radiographs, such as panoramic, periapical, and bitewings [20]. Histological examination is considered a reliable method to identify pulp stones and can detect a significantly greater number of pulp stones. However, this method is limited due to its invasive characteristic, and it is worth noting because pulp stones may not be detectable when limited sections are taken from each sample [20]. 

The findings of our study differ from research conducted on the Saudi subpopulation using CBCT [19]. In that study, they found pulp stones in 218 patients (50.93%) and 398 teeth (13.34%), which is higher than we observed. Another study by da Silva et al. [21] reported a rate of pulp stones in 122 patients (31.9%), which is higher than our study, and 269 teeth (9.5%), which is similar to our study. Similarly, Rodrigues et al. [1] found an incidence of pulp stones in the population they studied using CBCT, with 99 patients (55%). Both previous studies were conducted on the Brazilian population. Interestingly, our findings did not find any evidence of a correlation between Indian, Malizan, and Chinese populations regarding the occurrence of pulp stones [20]. The incidence was higher than the findings observed in our study [20]. This is likely due to differences in ethnic background and geographic location.

In contrast to our research, Al-Shammary and Al-Naznan identified a higher prevalence of pulp stones (859, 10.2%) in the teeth of a Saudi Arabian population using bitewing radiographs [17]. Hamasha and Darwazeh [22] discovered a higher occurrence of pulp stones in the Jordanian population compared to our study. They observed that 1006 (51%) of patients and 1006 (22%) of teeth in Jordanians had pulp stones [22], and Ranjitkar, Taylor, and Townsend [23] found a higher incidence of pulp stones in Australians compared to our research; they reported that 100 (46.1%) of individuals and 333 (10.1%) of teeth had pulp stones. This study's outcomes may be inconsistent with previous research due to several factors, including variations in methodology, ethnicity, age, and sample size.

In our findings, pulp stones were found more frequently in females than males. However, this finding is inconsistent with the results of earlier investigations conducted by Hamasha and Darwazeh [22] and Baghdady et al. [24], which found a higher occurrence of pulp stones in males than females, which conflicts with the findings of our study. In line with our findings, most of the literature suggests a higher prevalence of pulp stones in females than in males [17,18,25-27]. The current study identified a significant distinction between the maxillary and mandibular arches in addition to the left and right sides; this contrasts with the findings of da Silva et al. [21], Kannan et al. [20], and Hamasha and Darwazeh [22]. Our findings are consistent with earlier research in the literature that has indicated a significant discrepancy in the frequency of pulp stones in both arches and sides [17,18,22,23,27]. The present study demonstrated a higher prevalence of pulp stones in molars, which could be explained by their larger size, high chewing force, and high blood supply; all these factors contribute to a higher likelihood of calcification precipitation [27]. Also, the present study found a higher occurrence of pulp stones in teeth affected by caries, restorations, and periodontal disease than in intact teeth. However, according to Tamse et al. [18], Baghdady et al. [25], Ravanshad et al. [28], and Gulashi et al. [29], there was no significant difference in the prevalence of pulp stones and other risk factors.

The pulp stone increases with the patient's age, and some studies indicated that 90% of patients older than 51 have pulp stones. However, in our study, the prevalence of pulp stones in patients older than 51 can be much lower than in most previous studies [30]. This disagreement is probably due to our study's sample size and distribution, as when the CBCT images were collected, the patients were selected randomly without considering the patient's age. A sample with equal distribution is needed to conduct a study comparing the pulp stone with patient age, which means an equal number of patients in each age group need to be collected.

The limitation of the current study is that the result did not represent the whole population of Saudi Arabia because it was based on only one center. In order to ensure more reliable results, it is recommended that further studies involve multiple centers. The sample size was more extensive than some of the referred studies; however, a larger sample size is needed to obtain more comprehensive and accurate data. The equal distribution of the sample with age is necessary to ensure an equal distribution of the sample across different age groups.

## Conclusions

Understanding the prevalence and distribution of pulp stones is crucial for dentists and endodontists, as it assists practitioners in devising an appropriate treatment plan for affected teeth that require root canal therapy. One-fourth of the Madinah population was confirmed to have pulp stones, with a higher incidence in molars, caries, and restored teeth. No difference was found between its occurrence and gender. The high prevalence is exhibited in individuals between 45 and 54 years old. However, further studies with equal patient distribution are needed to confirm this observation.

## References

[1] Rodrigues V, Scamardi I, Schacht Junior CF, Bortolotto M, Manhães Junior LR, Tomazinho LF, Boschini S (2014). Prevalence of pulp stones in cone beam computed tomography. Dental Press Endodontics.

[2] Langeland K, Rodrigues H, Dowden W (1974). Periodontal disease, bacteria, and pulpal histopathology. Ora Sur Ora Med Ora Path.

[3] Sener S, Cobankara FK, Akgünlü F (2009). Calcifications of the pulp chamber: prevalence and implicated factors. Clin Oral Investig.

[4] BE G, JO PL (1956). Histogenesis and histochemistry of pulpal calcification. J Dent Res.

[5] Goga R, Chandler NP, Oginni AO (2008). Pulp stones: a review. Int Endod J.

[6] Arys A, Philippart C, Dourov N (1993). Microradiography and light microscopy of mineralization in the pulp of undemineralized human primary molars. J Oral Pathol Med.

[7] Nanjannawar GS, Vagarali H, Nanjannawar LG, Prathasarathy B, Patil A, Bhandi S (2012). Pulp stone--an endodontic challenge: successful retrieval of exceptionally long pulp stones measuring 14 and 9.5 mm from the palatal roots of maxillary molars. J Contemp Dent Pract.

[8] Jannati R, Afshari M, Moosazadeh M, Allahgholipour SZ, Eidy M, Hajihoseini M (2019). Prevalence of pulp stones: A systematic review and meta-analysis. J Evid Based Med.

[9] Estrela C, Decurcio DA, Rossi-Fedele G, Silva JA, Guedes OA, Borges ÁH (2018). Root perforations: a review of diagnosis, prognosis and materials. Braz Oral Res.

[10] Tang L, Sun TQ, Gao XJ, Zhou XD, Huang DM (2011). Tooth anatomy risk factors influencing root canal working length accessibility. Int J Oral Sci.

[11] Parekh S, Kyriazidou A, Bloch-Zupan A, Roberts G (2006). Multiple pulp stones and shortened roots of unknown etiology. Oral Surg Oral Med Oral Pathol Oral Radiol Endod.

[12] Bains SK, Bhatia A, Singh HP, Biswal SS, Kanth S, Nalla S (2014). Prevalence of coronal pulp stones and its relation with systemic disorders in northern Indian central punjabi population. ISRN Dent.

[13] Edds AC, Walden JE, Scheetz JP, Goldsmith LJ, Drisko CL, Eleazer PD (2005). Pilot study of correlation of pulp stones with cardiovascular disease. J Endod.

[14] Jena D, Balakrishna K, Singh S, Naqvi ZA, Lanje A, Arora N (2018). A retrospective analysis of pulp stones in patients following orthodontic treatment. J Contemp Dent Pract.

[15] Zeng J, Yang F, Zhang W, Gong Q, Du Y, Ling J (2011). Association between dental pulp stones and calcifying nanoparticles. Int J Nanomedicine.

[16] Srivastava KC, Shrivastava D, Nagarajappa AK (2020). Assessing the prevalence and association of pulp stones with cardiovascular diseases and diabetes mellitus in the Saudi Arabian population—a CBCT based study. Int J Environ Res Public Health.

[17] Al-Nazhan S, Al-Shamrani S (2011). A radiographic assessment of the prevalence of pulp stones in Saudi adults. Saudi endodontic journal.

[18] Tamse A, Kaffe I, Littner M, Shani R (1982). Statistical evaluation of radiologic survey of pulp stones. Journal of Endodontics.

[19] Patil SR, Ghani HA, Almuhaiza M, Al-Zoubi IA, Anil KN, Misra N, Raghuram P (2018). Prevalence of pulp stones in a Saudi Arabian subpopulation: a cone-beam computed tomography study. Saudi Endodontic Journal.

[20] Kannan S, Kannepady SK, Muthu K, Jeevan MB, Thapasum A (2015). Radiographic assessment of the prevalence of pulp stones in Malaysians. J Endod.

[21] da Silva EJ, Prado MC, Queiroz PM, Nejaim Y, Brasil DM, Groppo FC, Haiter-Neto F (2017). Assessing pulp stones by cone-beam computed tomography. Clin Oral Investig.

[22] Hamasha AA-H, Darwazeh A (1998). Prevalence of pulp stones in Jordanian adults. Ora Surg Ora Med Ora Path Ora Radi Endo.

[23] Ranjitkar S, Taylor JA, Townsend GC (2002). A radiographic assessment of the prevalence of pulp stones in Australians. Aust Dent J.

[24] Yaacob H, Hamid JA (1986). Pulpal calcifications in primary teeth: a light microscope study. The Journal of pedodontics.

[25] Baghdady VS, Ghose LJ, Nahoom HY (1988). Prevalence of pulp stones in a teenage Iraqi group. Journal of endodontics.

[26] Turkal M, Tan E, Uzgur R, Hamidi M, Colak H, Uzgur Z (2013). Incidence and distribution of pulp stones found in radiographic dental examination of adult Turkish dental patients. Ann Med Health Sci Res.

[27] Sisman Y, Aktan AM, Tarim-Ertas E, Ciftçi ME, Sekerci AE (2012). The prevalence of pulp stones in a Turkish population. A radiographic survey. Med Oral Patol Oral Cir Bucal.

[28] Ravanshad S, Khayat S, Freidonpour N (2015). The prevalence of pulp stones in adult patients of Shiraz Dental School, a radiographic assessment. J Dent (Shiraz).

[29] Gulsahi A, Cebeci AI, Ozden S (2009). A radiographic assessment of the prevalence of pulp stones in a group of Turkish dental patients. Int Endod J.

[30] Udoye C, Sede M (2011). Prevalence and analysis of factors related to occurrence of pulp stone in adult restorative patients. Ann Med Hea Sci Res.

